# The largest reported intrathoracic lipoma: a case report and current perspectives review

**DOI:** 10.1186/s13019-019-1030-8

**Published:** 2019-12-11

**Authors:** Mohammed Aldahmashi, Abdalmotaleb Elmadawy, Mahmoud Mahdy, Mohamed Alaa

**Affiliations:** 1 Surgery Department, Thamar University, Dhamar, Yemen; 2 Departments of Cardiac and Thoracic Surgery, PAAM Cardiac Center, Central Arar Hospital, Arar, Saudi Arabia; 3 Department of Cardiothoracic Surgery, Suez Canal University, Ismailia, Egypt

**Keywords:** Lipoma, Intra-thoracic, Fibrolipoma

## Abstract

**Background:**

The huge size intrathoracic lipomas are very rare. Few cases have been reported worldwide. To our knowledge, this presented case is one of the few cases reported. Here we report a single case as very huge intrathoracic lipoma compressing the right lung and displacing the diaphragm and liver downward. It has been managed by right posterolateral thoracotomy and complete excision, with excellent outcome.

**Case presentation:**

A 32-year-old male presented with a symptomatic right intrathoracic mass, which was confirmed to be a lipomatous tumor using computed tomography. A penduculated tumor originating from the mediastinal pleura was resected through the conventional right posterior thoracotomy. Pathological examination indicated a diagnosis of fibrolipoma.

**Conclusion:**

The tumor was symptomatic and relatively huge when detected during a medical checkup. This enabled the successful tumor resection via conventional thoracotomy approach. Although intrathoracic lipomas are histologically benign, careful observation and follow-up are crucial due to the possibility of recurrence.

## Introduction

The huge size intrathoracic lipomas are very rare. Few cases have been reported worldwide. To our knowledge, this presented case is the fourth reported [1]. Herein we report a single case as very huge intrathoracic lipoma (25 × 20 × 10 cm) compressing the right lung up and displacing the diaphragm and liver down which has been managed by right posterolateral thoracotomy and complete excision, with excellent outcome.

### Case history

**Fig. 1:**
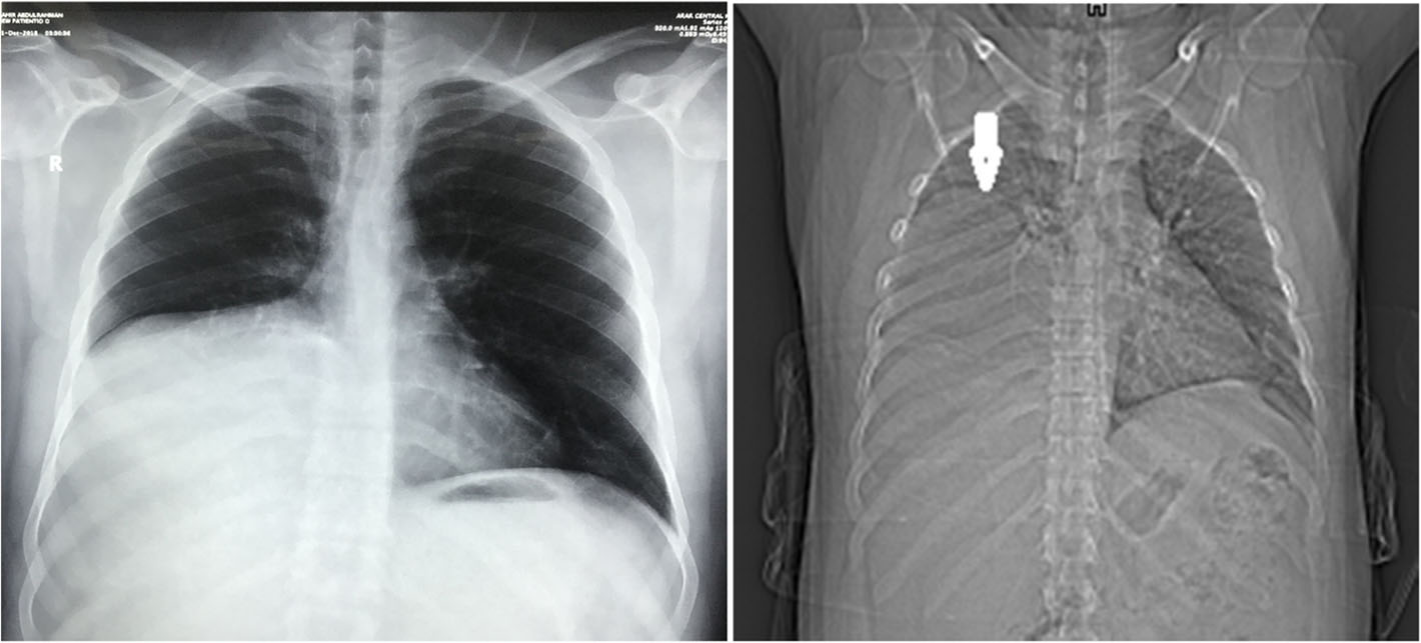
Chest X Ray showed an apparent huge mass occupying most of the right hemi-thorax obscuring lung field

A 32-year-old gentleman working as a hair dresser. His body weight is 100 kg, height 168 cm, Body Mass Index (BMI) was 35.43. Ex-smoker with a long history of smoking and long-standing history of chronic dry cough and shortness of breath but with no weight loss or hemoptysis. He had been managed as case of chronic obstructive pulmonary disease (COPD). He had history of surgical resection of multiple subcutaneous lipoma from the anterior abdominal wall. Recently, he was orthopneic with dull aching pain in the right lower chest to the right hypochondrial region. He was managed as a chest infection case then persistence of pain and dyspnea mandated performing the chest X Ray (CXR); it showed an apparent huge mass occupying most of the right hemi-thorax obscuring lung field (Fig. 1). Also, it showed how much the mass is moving upwards upon lying flat; producing profound lung compression.

**Fig. 2:**
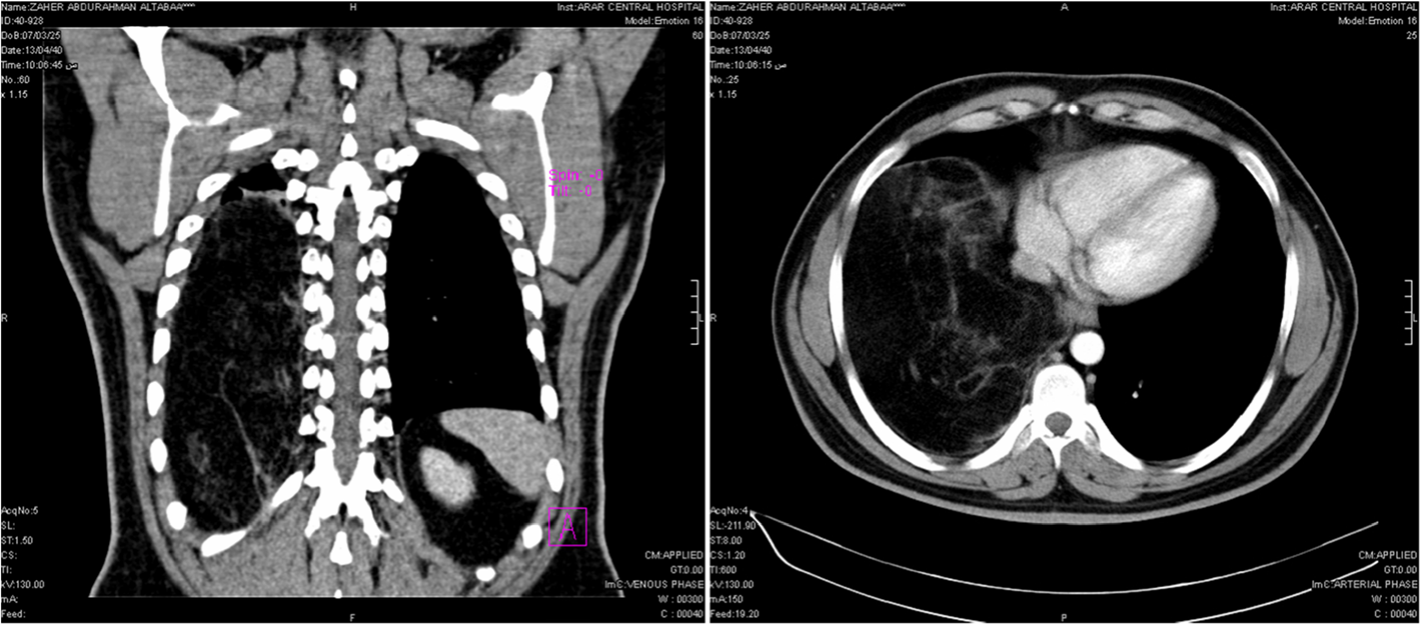
CT scan chest with contrast showing a well-defined huge intrathoracic mass

The patient was referred to our unit for surgical management. No history of trauma and examination was revealed absent breath sounds in the lower two-thirds of his right chest. Computed tomography (CT) scan with contrast of the thorax and upper abdomen (Fig. 2) was performed that showing a well-defined huge mass with soft tissue density (fatty pattern), not enhanced after intravenous contrast injection, with intact adjacent structure, located in the right hemi-thorax compressing the whole lung upward and displacing the diaphragm downward. There were no enlarged mediastinal or hilar lymph nodes. Spirometry revealed an obstructive pattern with a forced expiratory volume in 1 s (FEV_1_) of 1.6 L (54% predicted), forced vital capacity (FVC) 2.3 L (61% predicted) and an actual FEV_1_/FVC ratio of 70%. Blood tests were normal with a normal white cell count, hemoglobin, C-reactive protein (CRP) and erythrocyte sedimentation rate (ESR). An electrocardiogram showed normal sinus rhythm with iso-electric pattern.

**Fig. 3:**
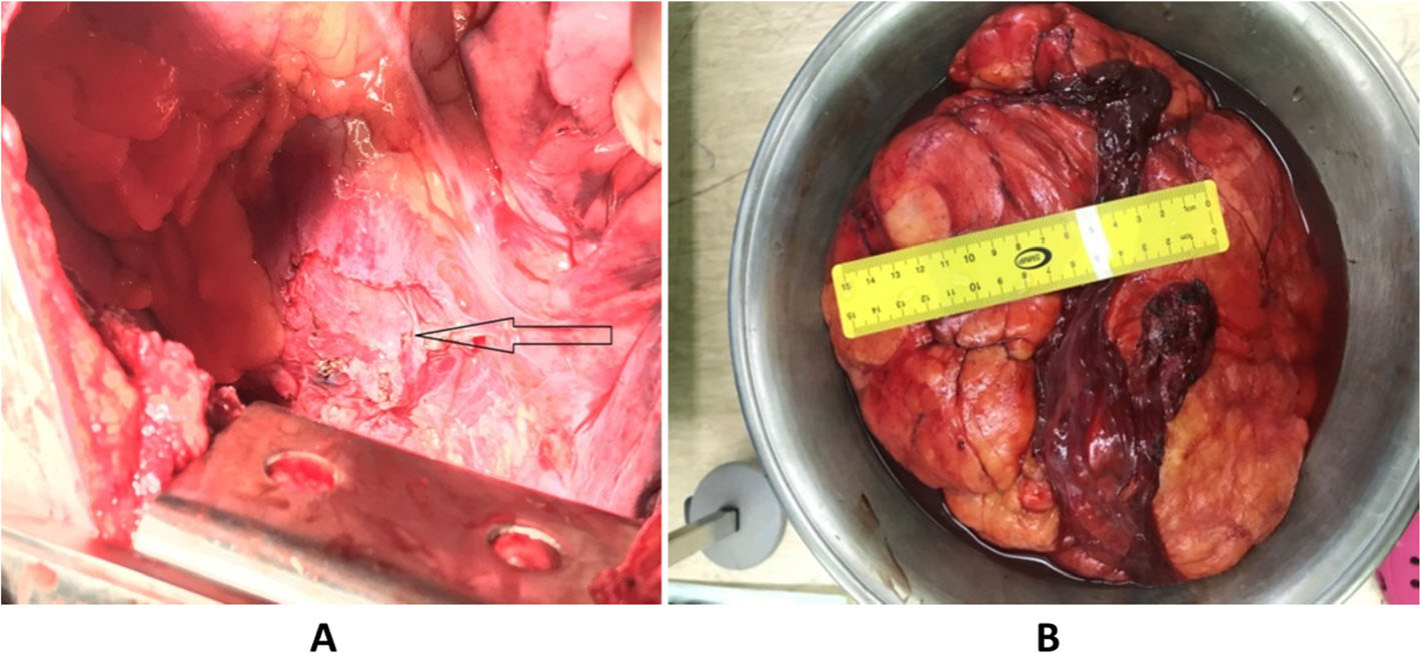
(**a**&**b**): R0 resection and the operative specimen

**Fig. 4:**
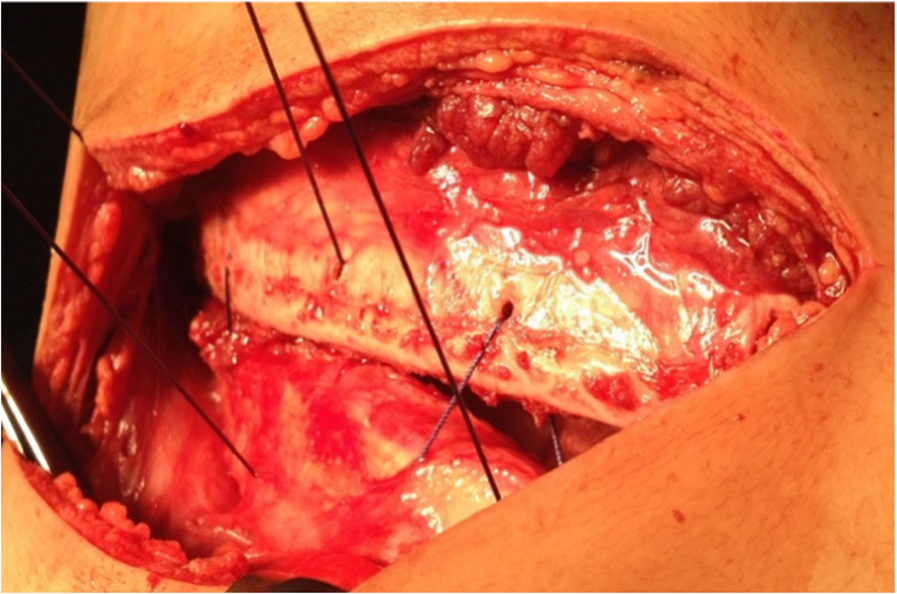
Intracostal suture with drilling

Surgery was performed using posterolateral thoracotomy, single lung ventilation and epidural analgesia. Huge sized, lobulated mass, yellowish, smooth surface, soft in consistency with small areas of hard components in its lower part and occupied most of the pleural space. This intrapleural lipoma attached with one vascular pedicle to the mediastinal pleura just above the right hilum required clipping by a multi-firing clip applier which facilitated the controlling of the pedicle as the mass obliterating the space. Blunt and electrocautery dissection performed for the minimal adhesion to the surrounding are required. A complete excision of this benign tumor (Fig. 3a) took place and subsequently the right lung is fully expanded and inflated. The mass measured 25 × 20 × 10 cm and weighed about 3500 g (Fig. 3b). The chest wound closure done in multi-layers fashion using intracostal suture with drilling (Fig. 4) this technique is precluding possible compression over the intercostal neurovascular bundle by the suture material and minimizing the post-operative pain significantly.

The postoperative course was uneventful, and the patient discharged home on the 6th postoperative day with a normal Chest X Ray.

**Fig. 5:**
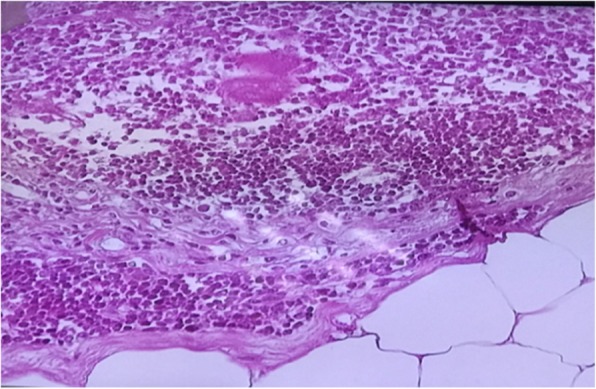
Rare Mixed Bengin Tumour: fibrolipoma Tumor; Exuberant lymphoid Nodular Hyperplasia with thymomatous differentiation

The Histopathlogical examination (Fig. 5) revealing fibrolipomatous benign tumor; Exuberant lymphoid nodular hyperplasia with thymomatous differentiation. The growth formed of variegated cellular elements with prominent sizable multilocular lobules that were encompressed of exuberant proliferated mature adipocytes encircled by thick fibrous banads. Infilterated by dense sizable lympoid follicles with germinal centers, calcifications, ischemic infarctions and mixed inflammation. No evidence of atypical mitosis or invasive malignancy could be seen.

**Fig. 6:**
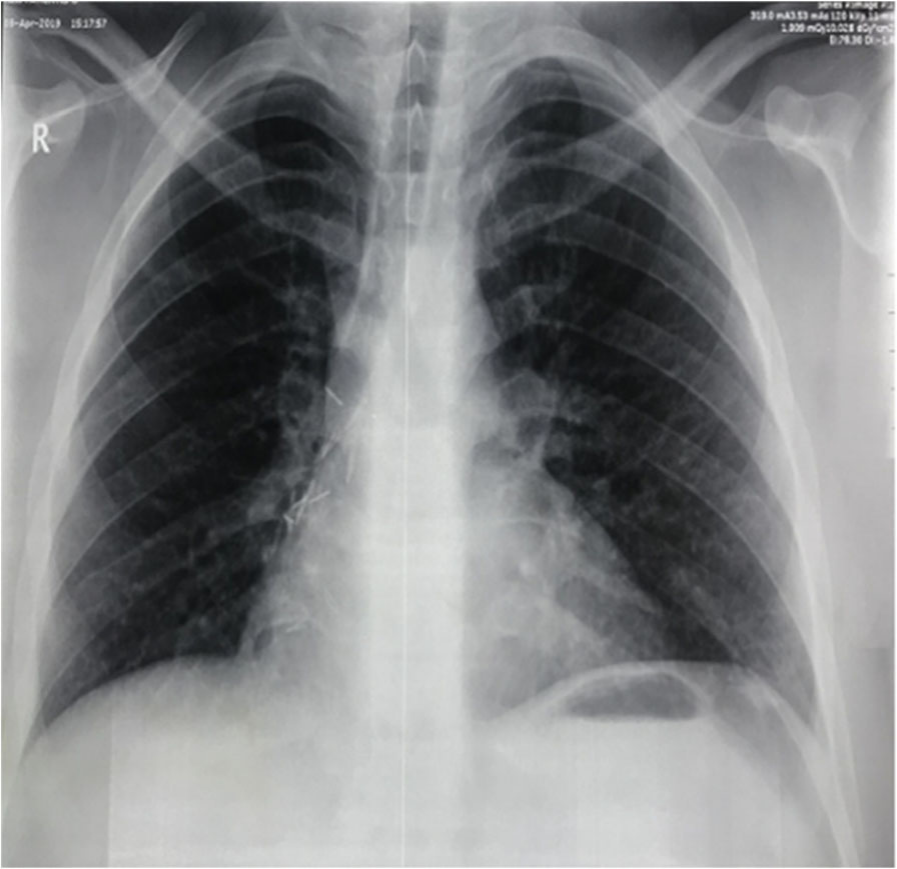
Normal CXR on 6 months follow-up

The postoperative course was uneventful and the patient discharge home on 6th postoperative day with normal Chest X Ray. Post discharge, his physical capacity and respiratory status improved dramatically, the walking distance is more than 5 km daily. No evidence of recurrence and normal CXR after 6 months of follow-up shown in (Fig. 6).

### Surgical perspectives for intra-thoracic lipomas

Since they were first described by Abbott and Webb in 1935 [2], Lipomas are classified as benign tumors that develop from adipose tissues at any site all over the body, they may contain mesodermal components other than adipocytes, including varying proportions of fibrous tissues and blood vessels [1]. It is the most frequent adult benign tumors (account for 20% of all benign soft-tissue tumors; though, fibro-lipomas are rare and only account for 0.03% of all) [1]. Intrathoracic lipoma may raise from the mediastinal fat covering the mediastinal pleura as in our case, or from the parietal pleura and its extension either intra- or extra-thoracic (i.e., Dumbbell-shaped lipoma) [3]. The pedicle of some lipomas may rise from the intercostals space [4, 5]. Mediastinal lipoma is rare and usually found in the anterior and seldom arise in posterior mediastinum [5]. Herein, we report one of the cases of huge intrathoracic lipomas that were successfully resected during conventional thoracic surgery.

Intrapleural and intrathoracic lipomas can enlarge with time and lead to pulmonary compressive symptoms such as cough, chest pain, dyspnea or a sensation of thoracic tightness, and compressive effect on the lung and diaphragm as in current case. An episode of chest pain, could be explained by repetitive episodes of fat necrosis [6] or due to pressure effect in huge size lipoma.

The first known complication is adjacent organ’s compression, these lipomas having significant volumes with an obstructive syndrome [5]. Large lipomas can induce lung compression and displacement of the surrounding structures as observed in our case it was displacing the right lung upwards and the diaphragm and the liver away down. Some authors have even reported compressing effect on the mediastinal structure [7]. Mediastinal lipoma typically grows very slowly, and the presenting symptoms are often related to a direct compression effect. The symptoms may include dysphagia, retrosternal compression and arrhythmia [5].

In current case the patient is overweight as his BMI was 35.43; other studies did not show any relation to obesity. Most of the reported similar cases were initially incidentally discovered by performing CXR as a soft tissue mass. CT-scan with contrast enhancement deemed as an essential tool to clearly define the relation of the mass to the adjacent structures (exhibit attenuation of fat of approximately − 100 HU on CT images), in our case the plain CT was non-conclusive but with enhanced contrast there is clear plane of cleavage and with an adipose density. The CT scan allows the attenuation values of fat density to be assessed and help determine the origin and extension, as well as the involvement of adjacent organs [1].

Additionally, magnetic resonance imaging (MRI), particularly with fat saturation, is supportive for assessing the lipomatous nature of the tumor. Furthermore, MRI helps in distinction between lipomas and well-differentiated liposarcomas based on margins, signal homogeneity, and septa or nodules [8]. Positron Emission Tomography (PET) scanning may also be an objective and useful modality for preoperatively evaluating tumors involving adipose tissue [1].

Lung malignancy especially in our case the pulmonary liposarcomas is the most important differential diagnosis in most unexplained CXR lesions particularly among heavy smokers. However, it is also important to consider benign disorders and intrapleural lipoma particularly when the lesion is with fatty pattern on CT. This can potentially avoid needless invasive investigations and alleviate patient anxiety [9]. CT scanning is a very helpful tool in clinical diagnosis, but surgical resection, with thoracotomy or VATS, remains a valuable procedure for establishing a firm diagnosis and complete excision [1, 10], some advocate the needle biopsy if the radiological diagnosis is not clear. Liposarcomas of the pleura are very rare tumors diagnosed mainly via CT-scans with lipomas being the main differential diagnosis. The challenge of diagnosis remains in identifying its origin and it needs careful inspection from the surgeon before resection [11].

In our case, despite the huge size of the tumor and there were minimal adhesions to the pericardium, lungs and diaphragm, and the phrenic nerve is intact, the diaphragm regains its normal contour and position after resection of the tumor. The origin is obviously from the mediastinal pleura just above the lung hilum with single wide vascular pedicle. The presence of extensive adhesion could be due to previous drainage procedures [1, 7].

The management of pleural lipomas remains controversial. Once the Intrathoracic lipomas are detected should be surgically resected, because the possibility of liposarcoma occurrence and/or infiltrating development of the tumor cannot be excluded preoperatively [12], besides the high probability of tumor recurrence after surgical resection [1].

Conservative clinical and radiological follow-up is often used in small asymptomatic lesions or those unsuitable or higher risks for surgery [13]. If compressive symptoms occur due to progressive enlargement then surgical resection should be considered in a fit patient. Because lipomas cannot be differentiated from malignant lesions, and they have invasive growth capability, surgery should be performed for diagnosis and treatment [14].

Open thoracotomy is the only option for total resection of the huge intrathoracic lipoma as in our case, although advancements in VATS have greatly reduced the morbidity rate of these benign tumors [1], especially if performed early on a small, uncomplicated adhesion-free tumor [13].

Thoracoscopic resection, in mediastinal cases, is generally considered to be a safe approach [1]. When video-assisted thoracoscopic surgery is indicated for the resection of an enormous tumor, there are two important aspects to keep in mind; the first is the nature of the neoplasm. If direct invasion of any vital organs is strongly suspected, such a procedure may carry additional risks and a change to open thoracotomy may be required. The second is the consistency as well as the size of the neoplasm. When the tumor is very soft, a small incision is sufficient for extraction even if the overall size of the neoplasm is very large [5]. In our case we couldn’t utilize the VATS as diagnostic tool because the non- invasive modality defined it clearly as resectable benign tumor and due to its huge size it is impossible to remove it via VATS.

## Conclusion

The tumor was symptomatic and relatively huge when detected during a medical checkup. This enabled the successful tumor resection of via conventional thoracotomy surgery. Although intrathoracic lipomas are histologically benign, careful observation and follow-up are crucial due to the possibility of recurrence.

## Supplementary information

**Additional file 1.** Informed consent

## Data Availability

All supporting data are available with the corresponding author.

## Abbreviations

BMI: Body mass index
COPD: Chronic obstructive pulmonary disease
CRP: C-reactive protein
CT: Computed tomography
CXR: Chest X Ray
ESR: Erythrocyte sedimentation rate
FEV_1_: Forced expiratory volume in 1 s
FVC: Forced vital capacity
HU: Hounsfield units
MRI: Magnetic resonance imaging
PET: Positron emission tomography
VATS: Video-assisted thoracic surgery
