# Supplementary material for: The largest reported intrathoracic lipoma: a case report and current perspectives review
Source: J Cardiothorac Surg. 2019 Dec 11;14:215. doi: 10.1186/s13019-019-1030-8 (PMC6907183; doi:10.1186/s13019-019-1030-8)
Supplement: Supplementary file 1 — Additional file 1. Informed consent [file 13019_2019_1030_MOESM1_ESM.pdf]

اقرار

أنا الموقع أدناة زاهر عبدالرحمن الطباع أوافق للدكتور محمد ناصر الدهمشي استشاري جراحة القلب والصدر على استخدام كل مايتعلق بحالتي المرضيه وكذلك العملية التي أجراها لي من وثائق ومعلومات شخصيه وصور وفيديو في النشر والبحث العلمي.

I,m agreed for publishing of my personal informations and doucments about my disease and the operation have been done for me by Dr. Mohammed Aldahmashi

الاسم : زاهر عبدالرحمن الطباع

التوقيع:

التاريخ: ٢٢ ٧ ٢٠١٩
